# The Real-World Problem of Care Coordination: A Longitudinal Qualitative Study with Patients Living with Advanced Progressive Illness and Their Unpaid Caregivers

**DOI:** 10.1371/journal.pone.0095523

**Published:** 2014-05-02

**Authors:** Barbara A. Daveson, Richard Harding, Cathy Shipman, Bruce L. Mason, Eleni Epiphaniou, Irene J. Higginson, Clare Ellis-Smith, Lesley Henson, Dan Munday, Veronica Nanton, Jeremy R. Dale, Kirsty Boyd, Allison Worth, Stephen Barclay, Anne Donaldson, Scott Murray

**Affiliations:** 1 King’s College London, Department of Palliative Care, Policy & Rehabilitation, Cicely Saunders Institute, London, United Kingdom; 2 University of Edinburgh, Centre for Population Health Sciences, Medical School, Edinburgh, United Kingdom; 3 University of Warwick, Health Sciences, Coventry, United Kingdom; 4 Institute of Public Health, The Primary Care Unit, Cambridge, United Kingdom; University of Stirling, United Kingdom

## Abstract

**Objectives:**

To develop a model of care coordination for patients living with advanced progressive illness and their unpaid caregivers, and to understand their perspective regarding care coordination.

**Design:**

A prospective longitudinal, multi-perspective qualitative study involving a case-study approach.

**Methods:**

Serial in-depth interviews were conducted, transcribed verbatim and then analyzed through open and axial coding in order to construct categories for three cases (sites). This was followed by continued thematic analysis to identify underlying conceptual coherence across all cases in order to produce one coherent care coordination model.

**Participants:**

Fifty-six purposively sampled patients and 27 case-linked unpaid caregivers.

**Settings:**

Three cases from contrasting primary, secondary and tertiary settings within Britain.

**Results:**

Coordination is a deliberate cross-cutting action that involves high-quality, caring and well-informed staff, patients and unpaid caregivers who must work in partnership together across health and social care settings. For coordination to occur, it must be adequately resourced with efficient systems and services that communicate. Patients and unpaid caregivers contribute substantially to the coordination of their care, which is sometimes volunteered at a personal cost to them. Coordination is facilitated through flexible and patient-centered care, characterized by accurate and timely information communicated in a way that considers patients’ and caregivers’ needs, preferences, circumstances and abilities.

**Conclusions:**

Within the midst of advanced progressive illness, coordination is a shared and complex intervention involving relational, structural and information components. Our study is one of the first to extensively examine patients’ and caregivers’ views about coordination, thus aiding conceptual fidelity. These findings can be used to help avoid oversimplifying a real-world problem, such as care coordination. Avoiding oversimplification can help with the development, evaluation and implementation of real-world coordination interventions for patients and their unpaid caregivers in the future.

## Introduction

Coordinating patient care is an important yet elusive global challenge and a substantial programme of work [1] to deliver improved coordination is underway in the US, the UK and Australia [2]. National recommendations have been issued to improve care coordination in the US and a coordination measurement atlas for the American healthcare community has been developed [3]. New Medicare payments that have been designed to improve coordination (estimated at $0.6 billion for primary care specialties) have been approved [4]. Similarly, coordination has been identified as a top priority for UK commissioners and considerable investments into coordination are being implemented. This includes technology solutions such as *Coordinate My Care,* which is being implemented across London [5], and keyworker roles [6]. Also, trials examining coordination interventions and studies to develop coordination measures have been conducted to address health care reform requirements and to improve care experiences and efficiency in Australia [7], [8].

This global need for improved coordination has the potential to escalate within the midst of a rapidly ageing population living with chronic illnesses, multiple morbidities [9], [10] and prevailing preferences to die at home [11]. Within Britain, these factors are forcing health and social care providers to integrate [12]. This shift towards integrated coordinated care is becoming especially important during a patient’s last year of life, when many professionals and services are involved in providing care [13]. In the last year of life, patients often present with multiple and complex needs [14] and this results in their care being provided by multiple providers and in multiple settings, including in the patient’s homes, in hospitals, outpatient clinics and GP surgeries [15]. Patients and families in this situation require care that is coordinated. That is, they require the deliberate organization of patient-centered care to optimize and integrate appropriate service delivery [1], both within and across care settings, and over time [16]. However, despite this need, coordination is often lacking at the end of life [8], [17], resulting in increased hospitalizations [18], missed appointments and reduced access to care [19], suboptimal clinical outcomes [17], fragmented care [20] and wasted time [19]. This is particularly evident for vulnerable groups, including older adults [21]. Systematic review data has also shown that when care is well coordinated, unpaid caregivers are more satisfied with the care that’s been provided for patients who have had a stroke [22] and those who required palliative care [17].

A large number of studies and systematic reviews have examined coordination interventions and a few interventions (for example, multidisciplinary teams and disease management interventions) have shown promise in reducing patients’ symptoms [17], and hospital admissions for patients with heart failure [18] and older adults [1]. However, no particular intervention has been identified as effective in addressing the problem of coordination [1]. The current limited theoretical understanding of coordination and the lack of research that has examined the views of patients and their unpaid caregivers is one explanation for this current lack of conclusive evidence. Indeed, experts agree that advances in coordination have largely been constrained by our limited theoretical understanding about coordination. Also, national developments have largely overlooked the value of patient and caregiver qualitative data to inform conceptual fidelity about care coordination. In fact, patient and caregiver perspectives are often missing when it comes to working out what coordination is and how best to measure it.

Our objectives are to address these gaps by developing a model of care coordination for patients and their unpaid caregivers living with advanced progressive illnesses in Britain, and by understanding the perspectives of patients and their unpaid caregivers regarding care coordination. Our research question was: “What is care coordination from the perspectives of patients living with advanced progressive illness and their unpaid caregivers?” Accordingly, we present a new model of coordination based on the views and experiences of patients and their unpaid caregivers.

## Methods

### Design

We used a longitudinal, multi-perspective qualitative methodology [23], [24] involving a case-study approach to allow for multiple and evolving perspectives regarding coordination and the integration of context-based factors related to each case in the final results. The study was conducted over a 26-month period commencing in 2010.

### Settings

In order to adequately explore coordination within Britain, data was collected from three contrasting settings (cases): 1) an urban combined acute admissions unit (CAU) that assesses and treats patients for up to 24 hours before hospital discharge or admission in Edinburgh (Scotland); 2) a general practice with working partnerships with care homes in the English Midlands (England); 3) three respiratory outpatient clinics (lung, chest and interstitial lung clinics) situated within an urban Academic Health Sciences Centre (AHSC) hospital in London (England). A core schedule of regular research meetings between sites was implemented to coordinate the research approach for all settings and to ensure consistency across and within local research teams.

### Consent

Informed written consent was obtained from all patients and unpaid caregivers by the field researchers (BM, VN, EE). This was gained before the first interview and prior to subsequent interviews. An informed consent form, approved by the ethics committee, was used for this purpose. All field researchers were experienced qualitative researchers who were employed as researchers to conduct this study. All were trained in the requirements of the study. BM, VN and EE had no prior relationship with the patients and or any of the unpaid caregivers.

The researchers attended multidisciplinary meetings and approached clinical staff in order to identify all eligible patients that might hold knowledge relevant to the research question. Once identified as eligible, staff would introduce the study to the eligible respondent and ask their permission for the researcher to approach them. Then, if suitable to the eligible respondent, the researcher approached the potential respondent to further explain the study and gain consent. The researchers outlined their role in the study and also explained the purpose of the study at the point of consent. Patients gave permission for their primary unpaid caregiver to be approached at this time or during subsequent interviews. Unpaid caregiver consent was also sought before each interview.

### Participants

Inclusion criteria for the patients were: a) attendance at the clinic as a registered patient with an advanced, long-term condition and or a new diagnosis of a progressive life-limiting illness; b) the clinician responsible for the patient needed to be able to answer “no” to the surprise question before the patient could be admitted to the study (the surprise question is: “Would you be surprised if this patient died in the next 6-12 months?”); c) the patient also needed to fulfill the Supportive and Palliative Care Indicator Tool (SPICT) criteria. The SPICT identifies patients living with one or more advanced, progressive, incurable condition, or those at risk of suddenly dying [25]; and d) the patient had to be in receipt of generalist care with or without specialist palliative care involvement. Generalist care was defined as encompassing all staff that work in health and social care without any specialist training in palliative care, including those that provide care for patients with end-of-life care needs. Examples of generalists for this study include general practitioners, district nurses, care staff and geriatricians. We acknowledge that generalist providers can also be considered as specialists and experts in their own right, but for the purpose of this study, we refer to them as generalists. Generalists were selected as our main focus, as they are the ones that deal with care coordination on a daily basis [24]. They also comprise the majority of the workforce in comparison to specialist palliative care providers, for example. Our definition and focus on generalists helped avoid only recruiting patients that were in receipt of specialist palliative care, which has been found to help improve coordination [17]. This distinction therefore helped widen the sampling of a broad range of coordination data in order to examine the nature of coordination in all its forms. Also, to ensure maximum variation, a sampling matrix guided purposive sampling of all patients based on: age, type of illness, types of need and social criteria. The exclusion criteria were that the patient: a) lacked the ability to consent to the research; b) was receiving care in the site while under police supervision; c) was unable to participate in the interview using English; d) was <18 years of age.

The inclusion criteria for the unpaid caregivers were that they were a case-linked caregiver and nominated by the patient as their primary caregiver. The exclusion criteria were that the caregiver was unable to consent to the research and or was <18 years of age.

### Data generation and management

**Figure 1:**
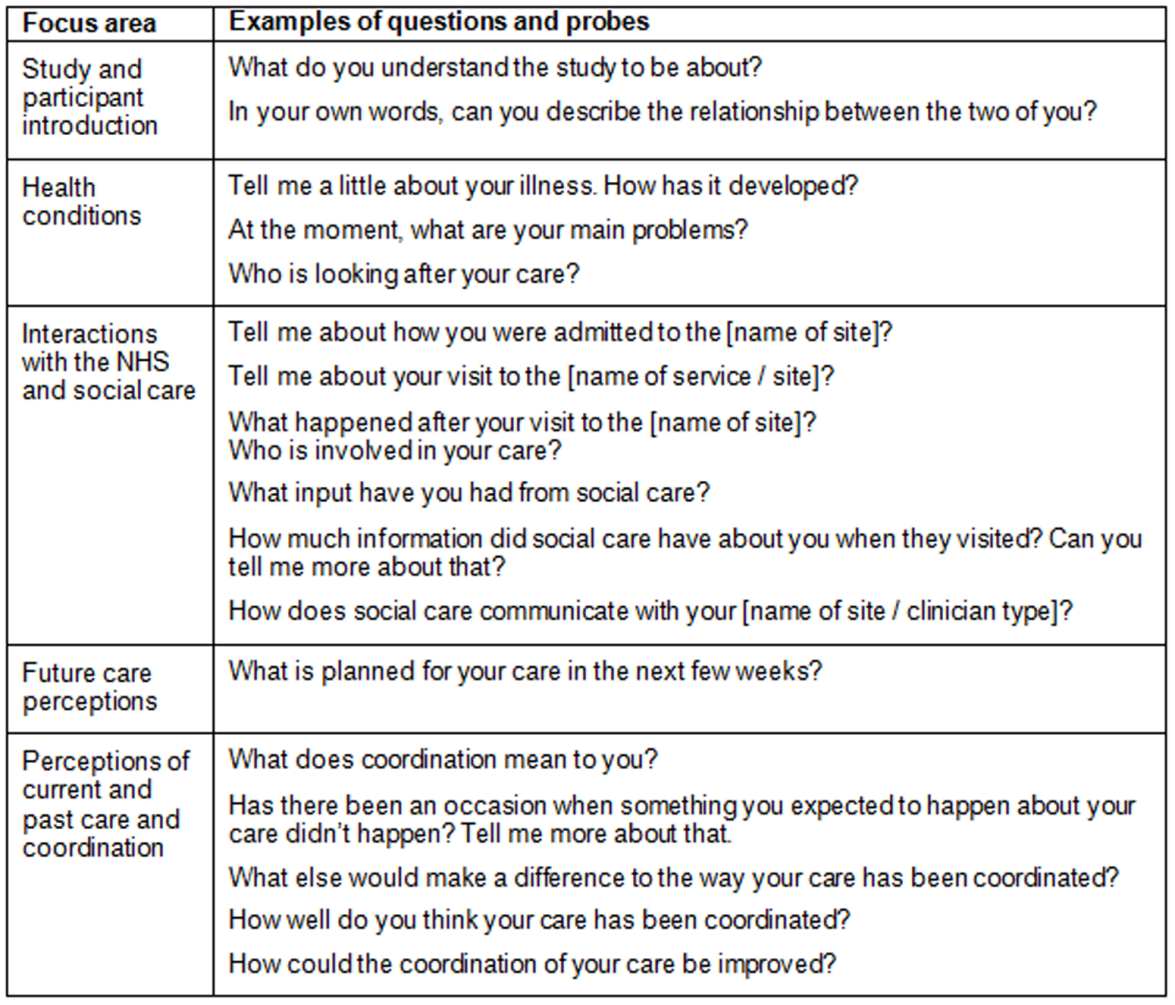
Outline of interview schedule and examples of questions and probes.

Up to three participant-led, semi-structured serial interviews were conducted at eight to 12 weekly intervals (where possible and acceptable to the patient). Each patient was followed for up to nine months or until their death. The interviews included a focus on experiences and definitions of care coordination, care experiences and challenges in achieving coordination. Participants were encouraged to reflect on their experiences of both health and social care aided by the use of a semi-structured interview guide, which was developed by the research team and refined within the field (Figure 1) [26]. Interviews were conducted at a place convenient for the participant, mostly in the research setting or the participant’s home. Each interview was transcribed verbatim and imported into NVivo for analysis.

In line with expert guidance [27], we used practical judgment and experience to determine a sample size that would enable deep and sufficient case-oriented analysis, which is an indicator of quality qualitative research. We also compared our sample size estimate with previously conducted multi-perspective, longitudinal qualitative research to ensure that our sample size was similar to existing robust studies [23]. The design and sample size was selected to allow for sufficient exploration of exceptional (deviant) cases and coordination over time within the context of serious illness. Furthermore, in line with expert guidance, the research ontology, design, sampling and the conceptual nature of our study was developed together in an integrated way to ensure logical generalizations from our findings to comparable settings [28].

### Analysis

Four overlapping analytical steps for each case (setting or site) were completed by three researchers (BD, CES, LH). First, open coding was used to comprehensively identify all and any concepts related to care coordination. Second, axial coding was completed to identify groups of concepts (also referred to as categories). Third, properties and dimensional ranges were identified (where data permitted), and then these were refined to illuminate the concept of coordination and to aid theoretical complexity [29], [30]. Analysis continued until data saturation was achieved [30], [31], which meant that analysis continued until no new insights emerged from the data. Four, inductive thematic analysis was used to identify underlying conceptual coherence across all cases in order to produce one coherent care coordination model. Cross-case analysis helped achieve this. During steps one through to three, patient interviews and caregiver interviews were analyzed separately and then combined to produce findings for each case at the end of step three. The three cases were combined through thematic analysis in step four. This sequence was used to avoid data fracturing [32].

#### Rigor

Analysis and credibility were aided through regular research meetings with the three research analysts. Visual mapping, diagrams and memos were used for reflexivity and transparency. Emergent and final analysis was checked to ensure logical and transparent representation of the data in our findings and to retain contextual accuracy [33]. In-vivo codes were used to stay close to participants’ perspectives. To ensure authenticity, all analysts, researchers and clinicians on the research team checked the final results. Many researchers in the research team were also clinicians. The research team included four GPs, a social worker, an occupational therapist, a music therapist, a specialist palliative care physician, two palliative care consultants and a senior clinical research nurse with management experience.

### Ethics statement

Ethical and local Research and Development Trust approvals were obtained for the study [Lothian Research Ethics Committee 10/s1102/17].

## Results

Fifty-six patients and 27 case-linked caregivers were followed through serial interviews with patients being interviewed alone (n = 29) or in patient-caregiver dyads (n = 27) (Table 1). Ninety interviews were conducted with patients in total and 60 interviews were conducted with unpaid caregivers (either alone or together with the patient). At the CAU, initially 40 patients consented to take part in the research. However, in the end, interviews were conducted with 20 patients and 11 unpaid caregivers from the CAU. Reasons for non-participation included: dying or becoming too ill, chaotic home conditions, readmission to hospital, changing their mind or simply not responding to contact after agreeing to participate. Sixteen patients and eight unpaid caregivers were interviewed from the general practice setting. Twenty patients and seven unpaid caregivers were interviewed from the outpatient clinic settings. In the outpatient setting, 42 patients were initially identified as potentially eligible by clinical staff, but after further reflection on the inclusion and exclusion criteria, 14 were not approached for recruitment. Consequently, 28 patients were approached by the researcher and ten declined participation. The top three reasons for declining were a) there was *“too much [going on] at the moment”* (n = 4), b) there was *“not much to say”* (n = 2) and c) the perceived lack of relevance of the study *“I only got chesty pains”* (n = 2). Therefore, 18 patients consented for the serial interviews in the outpatient setting. Overall, 29% of the patients recruited to our study had died by the end of the study.

**Table 1:** Patient ID, age, sex, main conditions, interview numbers, carer interviewed, carer’s relationship to patient, their sex, and the status of patient at the end of the study.

ID	Age	Patient Sex	Main conditions	Number of patient interviews	Carer interviewed	Number of carer interviews	Carer’s relationship to patient	Carer’s sex	Status of patient at end of study
CAU1	81	Male	COPD, heart failure (several heart problems)	1	No	0	–	–	Dead
CAU2	71	Female	Heart failure, renal failure, diabetes	1	Yes	2	Husband	Male	Dead
CAU4	85	Male	Heart failure, ischemic heart disease (IHD), mild dementia	3	Yes	3	Wife	Female	Alive
CAU5	86	Female	Pulmonary fibrosis, IHD, Paget's disease	2	No	0	–	–	Dead
CAU6	66	Female	Liver failure, diabetes, IHD	3	No	0	–	–	Alive
CAU8	56	Female	Neurological illness, polio, COPD, epilepsy, IHD	3	No	0	–	–	Alive
CAU13	89	Female	Unresponsive episodes, atrial fibrillation, hypertension, aortic stenosis, chronic vasculitis	3	Yes	3	Daughter	Female	Alive
CAU15	58	Male	Pancreatic cancer	1	Yes	1	Wife	Female	Dead
CAU17	75	Female	Diabetes, hypertension, depression, anemia	2	No	0	–	–	Dead
CAU20	75	Female	IHD	3	No	0	–	–	Alive
CAU25	71	Female	Multiple sclerosis	2	Yes	2	Husband	Male	Alive
CAU26	72	Female	Parkinson’s disease, asthma, pulmonary embolism	3	Yes	3	Husband	Male	Alive
CAU27	68	Male	Alcoholism, prostate cancer, peripheral vascular disease	3	No	0	–	–	Alive
CAU28	87	Male	Renal failure, diverticular disease, mild dementia, prostate cancer	3	Yes	4	Daughter	Female	Alive
CAU32	69	Male	Mitral valve disease, abdominal aortic aneurysm (AAA) repair, atrial fibrillation	1	No	0	–	–	Dead
CAU33	71	Male	Hypertension, atrial fibrillation, heart failure	3	Yes	2	Wife	Female	Alive
CAU34	85	Male	Hypertension, motor neuron disease, arthritis, asbestosis, peptic ulcer disease	1	No	0	–	–	Alive
CAU37	69	Male	Peripheral vascular disease, IHD, diabetes	3	Yes	2	Wife	Female	Alive
CAU39	60	Male	Multiple sclerosis	3	Yes	2	Mother	Female	Alive
CAU40	88	Female	Renal failure, metastatic melanoma	1	Yes	1	Son	Male	Dead
GP1	67	Male	Diabetes, Charcot’s arthropathy, cellulitis	3	No	0	–	–	Alive
GP2	79	Female	Diabetes, heart failure, osteoarthritis	3	No	0	–	–	Alive
GP3	79	Male	Renal failure, heart failure, IHD, hypertension, osteoarthritis	3	Yes	3	Wife	Female	Alive
GP4	82	Female	Lung cancer, stroke, IHD	2	Yes	2	Husband	Male	Dead
GP5	56	Male	Pulmonary fibrosis, cerebral aneurysm, hyperlipidemia	2	No	0	–	–	Dead
GP6	82	Male	Renal failure, heart failure, anemia, osteoarthritis	3	Yes	3	Wife	Female	Alive
GP7	92	Male	Respiratory failure, heart failure, renal failure, osteoarthritis, blind (glaucoma)	3	Yes	3	Wife	Female	Alive
GP8	80	Male	Prostate cancer, mild dementia, osteoarthritis	1	Yes	1	Wife	Female	Dead
GP9	71	Male	Multiple sclerosis, osteoarthritis	3	No	0	–	–	Alive
GP10	73	Male	Prostate cancer, mild dementia, hypertension	2	Yes	2	Wife	Female	Alive
GP11	71	Female	Multiple sclerosis	3	No	0	–	–	Alive
GP12	67	Male	Peripheral vascular disease, renal failure, COPD	3	Yes	3	Wife	Female	Alive
GP14	41	Male	Metastatic melanoma	1	Yes	1	Wife	Female	Dead
GP15	81	Female	COPD	1	No	1	–	–	Alive
GP16	58	Male	Multiple sclerosis	2	No	0	–	–	Alive
GP17	90	Female	Stroke, osteoarthritis	1	No	0	–	–	Alive
Clinic1	61	Female	Lung cancer, metastatic to adrenal glands	3	Yes	3	Son	Male	Alive
Clinic2	68	Male	Lung cancer, emphysema (severe), breathlessness	3	Yes	1	Domestic partner	Female	Alive
Clinic3	76	Male	Lung cancer/adenocarcinoma, Asbestosis, CABG	3	No	0	–	–	Alive
Clinic4	82	Male	Lung cancer with leg metastasis, ischemic heart disease (IHD), CABG	1	No	0	–	–	Dead
Clinic5	54	Male	Lung cancer with bone metastasis	4	Yes	3	Daughter	Female	Dead
Clinic6	78	Male	Idiopathic pulmonary fibrosis, IHD, emphysema, breathlessness, pulmonary hypertension	2	Yes	2	Wife	Female	Alive
Clinic7	55	Female	Pulmonary embolism, dermatomyositis, breast cancer, hypothyroidism, very breathless	2	No	0	–	–	Alive
Clinic8	70	Male	COPD (asthma)	3	Yes	3	Wife	Female	Alive
Clinic9	90	Female	Lung cancer, COPD, osteoporosis, long standing back pain	2	No	0	–	–	Dead
Clinic10	72	Male	COPD, emphysema	3	No	0	–	–	Alive
Clinic11	59	Male	Lung cancer, with stomach, throat and bone metastasis	1	Yes	1	Domestic partner	Female	Dead
Clinic12	65	Male	Squamous cell lung cancer, breathless on exertion	3	No	0	–	–	Alive
Clinic13	69	Male	Lung cancer [with kidney metastases], COPD, emphysema	2	Yes	1	Domestic partner	Female	Dead
Clinic14	63	Male	COPD (severe)	3	No	0	–	–	Alive
Clinic15	66	Female	COPD	2	No	0	–	–	Alive
Clinic16	52	Female	COPD [emphysema/ bronchial bronchitis]	1	No	0	–	–	Alive
Clinic17	46	Male	Lung cancer (small cell) with brain metastasis	1	No	0	–	–	Alive
Clinic18	64	Female	Upper lobe lung cancer, type II diabetes, hyperthyroidism	1	Yes	1	Son	Male	Alive
Clinic19	74	Female	COPD	1	No	0	–	–	Alive
Clinic20	79	Male	Lung cancer, COPD, chronic asthma, heart failure	1	No	0	–	–	Alive

### Overall findings

**Figure 2:**
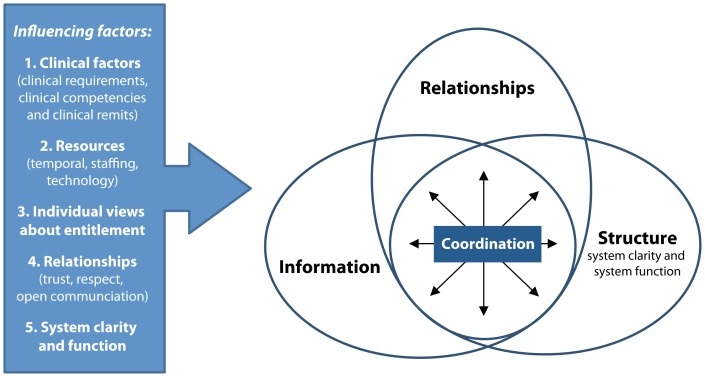
The coordination of generalist palliative care (CoG model).

Thirteen categories and 10 influencing factors were identified across the three cases (Tables S1–S3). Analysis of these primary case findings, including the categories (Table 2) and influencing factors (Table 3), resulted in one empirical model, which has three interactive elements and five types of influencing factors (Figure 2). The thematic analysis findings are presented here in the form of one coherent conceptual model called “The coordination of generalist palliative care involving advanced progressive illness” (or otherwise referred to as the *CoG model*).

**Table 2:** Categories from the thematic analysis of the primary findings from the three cases.

Core element	Category from primary findings from each case (setting, country)*
Quality, well-informed, caring staff working in partnership	Knowledge and engagement (CAU, Scotland); staff trust and efficiency (CAU, Scotland); high-quality care and staff with clear roles, relationships and adequate resources (General practice with care homes, England); standardized, automated, reliable, cohesive systems and services with a personal touch (General practice with care homes, England); responsive, logical and simple systems centered on patients and unpaid caregivers (General practice with care homes, England); unpaid caregivers and patients coordinate care, but at a cost (General practice with care homes, England); professional characteristics (Outpatient clinics, England); patient empowerment, knowledge and experience, and ways to avoid waste (Outpatient clinics, England); recognition of caregivers as coordinators (Outpatient clinics, England)
Knowledgeable and expert patients and caregivers who coordinate care, but at a cost	Staff trust and efficiency (CAU, Scotland); high-quality care and staff with clear roles, relationships and adequate resources (General practice with care homes, England); responsive, logical and simple systems centered on patients and unpaid caregivers (General practice with care homes, England); unpaid caregivers and patients coordinate care, but at a cost (General practice with care homes, England); patient empowerment, knowledge and experience, and ways to avoid waste (Outpatient clinics, England); recognition of caregivers as coordinators (Outpatient clinics, England)
Patient-centered, efficient, adequately resources, inter-connected, centralized, automated systems and services	Flexible and convenient care (CAU, Scotland); flexible and convenient care (CAU, Scotland); responsive, logical and simple systems centered on patients and unpaid caregivers (General practice with care homes, England); timely, traceable, accurate and useful information with consideration of the implications of this information on unpaid caregivers (General practice with care homes, England); interconnected service structures and IT systems (Outpatient clinics, England); recognition of caregivers as coordinators (Outpatient clinics, England)
Accurate, timely information communicated with consideration of patients’ and caregivers’ needs, circumstances, abilities	Knowledge and engagement (CAU, Scotland); responsive, logical and simple systems, centered on patients and unpaid caregivers (General practice with care homes, England); responsive, logical and simple systems, centered on patients and unpaid caregivers (General practice with care homes, England); timely, traceable, accurate and useful information with consideration of the implications of this information on unpaid caregivers (General practice with care homes, England); unpaid caregivers and patients coordinate care, but at a cost (General practice with care homes, England); professional characteristics (Outpatient clinics, England); recognition of caregivers as coordinators (Outpatient clinics, England)

* The detail in Tables S1–S3 informs the results presented in Table 2

**Table 3:** Results of the thematic analysis of the he ten influencing factors from the primary findings from each case.

Types of influencing factors	Influencing factor identified from each case (setting, country)*
Clinical	Transitions: defined as time points where a change in care delivery occurred or a transition to a new treatment needed to take place (CAU, Scotland); communication across settings (General practice with care homes, England)); staff focus, experience and working relationships (Outpatient clinics, England); care based on needs rather than diagnosis (Outpatient clinics, England)
Resources	Adequate resources (General practice with care homes, England); temporal constraints (for example, not having enough time to do your job) (General practice with care homes, England); personnel and temporal resources (Outpatient clinics, England); patients’ views and wishing to not burden the system or staff (Outpatient clinics, England)
Individual views about entitlements	Patients’ views and wishing to not burden the system or staff (Outpatient clinics, England)
Relationships	Communication across settings (General practice with care homes, England); staff focus, experience and working relationships (Outpatient clinics, England)
Characteristics of the health system	Communication across settings (General practice with care homes, England); management systems, efficiency and decisions (Outpatient clinics, England); system clarity (Outpatient clinics, England)

* The detail in Tables S1–S3 informs the results presented in Table 3.

### The CoG model

#### Essential ingredients

In the midst of advanced progressive illness, coordination is a shared complex intervention that comprises an essential mix of relational, structural and information elements.

The relational components involve relational work between health and social care staff, patients and their unpaid caregivers. “It was just like, they would come round, this is the strange woman or the, you know, and off. And that was it. You didn’t have time to ask them. And you felt as if you did ask them, you would be holding them back.” [PatientCAU008] “There’s lots of little things that need to be looked at that, but things that people [staff] take for granted, but they’re, they’re just automatically done by family members.” [CarerClinic001]

The structural components involve an emphasis on systems and services in relation to their clarity and function. “I got the best care, yeah, I certainly did at hospital [X]. What annoyed me, and it can’t be helped, but what annoyed me, I was getting lost, ‘cos one minute I was going to hospital [X] for stuff, I’d go ‘Right, I know I’m going to hospital [X]’. ‘No, hospital Y’. I’d go ‘What, hospital X? Hospital [Y]?’ ‘No, hospital Y’. ‘Oh come on’. ‘No, they haven’t got that course there so you’ve got to go to this one for this course’. ‘OK, fair enough. Where am I at?’ (laughs)” [PatientClinic014]

“Well I have always trusted the system to be there when you needed it to provide these magical supports from somewhere when there was a crisis. And it just doesn’t work that way — you have to chase, you have to know the routes to get things, and which buttons to push. I mean, my mum was saying, my brother [who works] at the [hospital], he was actually a Godsend, it was such a reassurance, having somebody on the medics side, which knew which buttons to push. He was able to kind of, help things along a wee bit, just by being there and knowing how the system works. He was almost taking on an advocacy role for us.” [CarerCAU020]

The information element stresses the timely provision of information and the importance of effective communication. “Very, very professional, someone who you’d say absolutely knows what he’s doing, knows how to speak to a patient in such a circumstance. Well it, for me, that was what was the possible scenario. But the way he spoke to me, the way he explained it, you couldn’t have asked for anyone better…” [PatientGP005] “…the care structure is, it just seems to be, it always seems to come down to communication, or lack of communication or misunderstanding and communication, so the communication’s not quite there…between organizations, between departments within their own organizations. So, for instance, the money’s going to Social Services, yes, we know it’s going there, we didn’t know the exact amount that was going there, we didn’t know the person who was allocated the money to look after wasn’t in the office, or in a different department, the communication between the social worker and that person is difficult…and it’s like the Blue Badge. The Blue Badge have got a letter from my mum’s nurse, it says everything in the letter, it’s quite obvious how important it is, but the Blue Badge is not going to happen because they haven’t got the award letter for the DLA, for the Disability Living Allowance. The reason they haven’t got the award letter for the Disability Living Allowance is ‘cos it hasn’t been sent out. Why hasn’t it been sent out? I don’t know. All I know is when I was at the hospital, the [staff member] said to me ‘You don’t need to do anything. It’s fine. We, I’ve done this form automatically for you, it will be a higher rate Disability Living Allowance from the [date].’ The higher rate of Disability Allowance, from what I’ve been told, hasn’t been paid up. There’s no award letter, because I haven’t got no award letter I can’t get the disabled badge. So it’s another thing that’s just made difficult.” [CarerClinic001] “Well, it’s coordination issues, like probably with me, it’s the different departments that I’m getting, what can I say? Getting attention from different departments, and it all coming to me and then I manage to relay something back to them, certainly with the tests I did, and certainly with the test trials, you know.” [PatientGP006]

Data showed that participants view coordination as a deliberate cross-cutting action involving high quality, caring and well-informed staff, patients and unpaid caregivers who must work together in partnership and across primary, secondary and tertiary settings, as well as across health and social care divides. Respondents shared that patients and unpaid caregivers may contribute substantially to the coordination of care, which, at times, is volunteered at a personal cost. *“I subsidise my mum to the tune of £1,000 out of my own pocket, so in addition to the amount of support that I’m putting on, I’m losing time, which I can’t put into running my own business, so I’m losing money there, and I’m subsidising my mum….So it does make an issue, it is an issue, it is an issue.” [CarerClinic001] “Because years ago I wouldn’t have said boo to anybody I mean really being honest, but I know now that you’ve got to, if you want something done you’ve got to stick up for yourself a bit now and of course particularly when it’s him. I probably don’t bother about myself but I’m always checking that he’s getting the right attention…” [CarerCAU004]*

Patients and unpaid caregivers explained that for coordination to occur it must be adequately resourced with efficient systems and services. *“The [staff member] has got too much work, yeah, that’s not my problem but he has too much work.” [CarerGP001]* They explained that coordination is facilitated through flexible and patient-centered care, characterized by accurate and timely information communicated in a way that considers patients’ and caregivers’ needs. *“I didn’t know granddad had been moved. My heart just was in my mouth, you walk in and you see this empty bed. I mean, somebody should have phoned, just to say, he’s been moved. And for it to happen three times, to be going in ‘where is he now?’ You know? And then the other thing I always think about hospitals is, when you go at visiting time, everybody seems to disappear, but that’s the only time a relative goes in. So, there is nobody to ask.” [CarerCAU028]* Patients’ and unpaid caregivers’ preferences, circumstances and abilities also need to be taken into account. *“Dr [X] used to send me a copy of anything he wrote. Every time I saw him, he would write a report saying, I’ve seen Mr X again, blah, blah, blah, he this, that and the other. And there’s a lot of technicalities in the letter about the rate of my absorption or expressing of oxygen and carbon, is it dioxide or monox…? Dioxide. And I don’t understand a word of it, ‘cos it’s all technical. But at the bottom of these letters, as you know, they say, if you don’t want to receive a copy of any communication, just let us know. And I told them three times, there’s no point, I don’t understand them (laughs) and it’s just wasting stamps and wasting money and wasting time for the National Health Service (NHS). But they keep on sending them. Now there you get put on computer and they’re automatically there in hospital X. So anything he writes about me goes on the computer. So if I go up there now and say ‘Oh I’m having an attack’ or ‘I’m, I’m desperate’ they will have everything on the computer.” [PatientClinic010]*

### Influencing factors

Five types of factors influence coordination: a) clinical factors, b) resources (time, staffing, and technological resources), c) individual views about entitlements, d) relationships, and e) characteristics of the health system (system clarity and function).

#### Clinical factors

Patients and unpaid caregivers shared that clinical factors (for example, their illness and the severity of their illness) influence their need for coordination. Their need for coordination therefore changes over time in response to clinical factors. For example, when moving from one setting to another (because of a clinical need), their need for coordination might increase. “The reality kicks in once someone’s home, which is where the follow up visits are so important.” [CarerCAU113] Similarly, more coordination may be required when their care or treatment changes in some way. Patients and unpaid caregivers also shared that the clinical roles and competencies of staff, individual approaches of staff and the professional roles/remits of staff influence the coordination of their care. All of these clinical factors combine to produce variance in care coordination. For example, participants shared that some staff are pro-active when it comes to coordinating their care, as they view coordination as an integral part of their role. “The doctor I saw said she would see that I got help to come home to and I wouldn’t get out [of hospital] until she got that sorted and she was quite right because I got somebody the next morning.” [PatientCAU005] Their input then naturally helps streamline the care process and coordinate care overall, and their effectiveness is aided by their overall professional and personal competencies. However, respondents also shared that some staff do not consider coordination at all, as they do not view it as being part of their job. They said that this sometimes results in sub-optimal experiences and fractured experiences, support and care. “Well, my suspicion is that they assume that someone else is communicating that information. And unfortunately, mum, and the generation that mum comes from, they don’t question as perhaps, vigorously as they should.” [CarerCAU013]

#### Resources

Resources also influence coordination. Patients and caregivers shared that their care was more likely to be coordinated when staff had sufficient time to coordinate care and when the clinical teams were adequately staffed. “I think the greatest problem though, nowadays, is they promise you help and because the shortages, because that is what’s happening, that they are not getting the help, you know, that they should get. And I think that’s the greatest problem, it is very sad, you know. Because, you know, they kept asking me, they kept telling me, the hospital, that I would, particularly at the early stages, about the help I would get. And it wasn’t there, I never got any help, I just had to get on with it.” [CarerCAU004] “And I notice it up here, you can phone, you know, if you feel it is not anything important, you can phone in and the doctor will talk to you or you can phone a nurse and they will talk to you. Please don’t make an appointment unless it is absolutely necessary. Well, what do you know is absolutely necessary? That’s my argument. I never send for a doctor, she’ll tell you — I could be practically dying before I’ll send for a doctor. And that’s mostly my problem. I have been rushed into hospital because I have left it too long, because I won’t bother them.” [PatientCAU020]

Respondents also highlighted the potential of technology to aid coordination. They suggested that technology could help automate follow-up appointment bookings. Technology could also help improve communication across social care and healthcare services. For example, technology could help when it comes to organizing transport for appointments, help coordinate discharge planning and help coordinate care that is provided in the patient’s home. “*I wouldn’t have thought it would be difficult to have a sort of audio signal on the computer…surely it could be flagged up…beep, beep, beep…maybe the bit about special transport or this bloke is violent or whatever else it is…” [PatientGP001]*

#### Individual views about entitlements

Individual views and expectations about care also influenced how their care was coordinated. Some shared that they wanted to avoid burdening the healthcare system and staff. In these instances, some patients and unpaid caregivers were prepared to “put up” with the standard of care they received. However, others described that they were aware of what they were entitled to and therefore they expected a certain level of care coordination. “I hate causing trouble but I had to argue with them this time that I wasn’t prepared to take him home, you’ve just to be different to your usual you know….I come from a social work background…I see so many isolated elderly people, who trust the system too much. Who aren’t able or don’t think they are allowed to challenge or even to ask the question: ‘Why is this happening?’” [CarerCAU113].

#### Relationships

The fourth factor influencing coordination was relationships. Patient and caregiver relationships with staff influenced coordination and relationships between staff influenced coordination. Patients and unpaid caregivers shared that they thought a collegial relationship, based on mutual trust and respect, was more likely to result in their care being coordinated. “There’s a lot of medication that I’m taking, that’s been changed since [going to hospital], that I hadn’t gone back to the GP. They say they’re sending it back but it never seems to come. I go down there a week later and they [the GP] don’t know anything about it…I’m going like an idiot to the doctor, telling me about, he doesn’t believe me that I am taking sixteen a day now.” [PatientClinic011] Their care may be less well coordinated when staff don’t respect each other as individuals or as professionals. “They don’t seem to communicate with each other, if you know what I mean. You’ll get one and they’ll say one thing and then the next one might not say anything, and you wonder what’s wrong, they’re not saying it, you know.” [PatientCAU005]

An open style of communication was said to help coordination, as it kept patients and unpaid caregivers informed about what was happening. An open style of communication also meant they would be involved in making decisions about their own care. *“Yes, they gave me a letter, they gave me a letter for the doctor and they gave me a letter for the district nurse, and they gave me a discharge letter, and I took them along that afternoon. Because when he, after he had gone home at half past two and he wanted to sleep and I said ‘are you alright?’ I waited for a wee while and he said he was fine, he was sleeping. So I said I’ll go along, walked along to the medical centre and handed them in, which of course was good because the district nurse arrived the next day.” [CarerCAU004]*

#### System clarity and function

System clarity and function was the fifth factor to influence coordination. Systems that functioned well and systems that were easy to understand made it easier for care to be coordinated. The inverse of this was also evident. That is, it was harder to get coordinated care if you couldn’t work out how the health and/or social system worked. It was also difficult to achieve coordination when there was a system breakdown or disruption of some sort. For example, coordination was hindered when information was not able to be shared between GPs, hospitals and pharmacies. “So we’re continually having to tell them, and the frustration of saying, ‘but surely you must now have that on records?’” [CarerCAU013] Also, it was harder for care to be coordinated when information couldn’t be shared between hospital staff and staff that provided care in the patient’s home. “And then my daughter will ring the GP about me, for something and they will say ‘Oh is he out?’ that sort of thing. I would say to them ‘Yes, I’ve been out three, four days. Didn’t you know?’ ‘Oh nobody’s told me’”. [PatientGP004] Clarity and optimal service delivery from service managers were also important, as this could impact upon the coordination of care for patients and their unpaid caregivers. “The point is, there are three supervisors… but…the other day we didn’t know who was coming [into our home] for two days…” [CarerGP007]

## Discussion

### Summary of findings

The main result of this study is a new conceptual model of care coordination, referred to as the CoG model, which is derived from patient and unpaid caregiver data. The model has five influencing factors and three key active ingredients: relational, structural and information elements. Our findings highlight the need for composite outcomes and generic measures that quantify the true multifaceted nature of coordination in evaluation studies. Our findings also help open up current discussions about which coordination activities should be linked with fiscal incentives. The role of patients and unpaid caregivers in relation to care coordination is also emphasized in our study, along with the value contribution they make to the coordination of care. Furthermore, our research highlights the challenges that lie ahead when it comes to researching the real-world problem of care coordination, which takes place in a complex health and social care system that caters for people with complex needs. Avoiding oversimplification of the real-world problem of care coordination can be useful when it comes to developing, evaluating and implementing coordination interventions for patients and their unpaid caregivers in the future.

### Comparison with existing coordination definitions and fiscal incentives

The multifaceted nature of our new CoG model broadly corresponds with existing care coordination definitions derived from clinical record data, observations and expert opinion data. However, in our COG model, relational and structural elements feature just as prominently as the information elements in existing models. This equivalence is not always prominent in existing models, which have mostly included an emphasis on the involvement of multiple participants, the need for knowledge and resources, an emphasis on information and the adequacy of services [1]. The relational details in the CoG model highlight not only the importance of the involvement of multiple stakeholders, but also the importance of how staff, patients and unpaid caregivers work together, how they relate to each other and also the personal qualities of the individual staff (for example, whether or not they genuinely care about the patient, as shown in the Tables S1–S3).

These findings mean that a fiscal incentive for tasks to cover transitional care management (for example, for patient discharge following an inpatient hospital admission, partial hospitalization, skilled nursing facility discharge or community mental health care discharge), like the one introduced by Medicare in the USA in 2013 (estimated at $0.6 billion [34]) to aid care coordination activity (codes: 99459 and 99496), may not adequately address the complexity of the problem of coordination. Even though the emphasis on primary care physicians and transitions for these new incentives is useful, our findings suggest that this will only partially address the problem of care coordination. When fiscal incentives are used they need to cover more than just this.

### A comparison of the CoG model with other models

Most models of coordination have been developed through theorizing [35] rather than arising from patient and unpaid caregiver data. Furthermore, many existing models have been theoretically adapted to fit coordination rather than being develop in relation to coordination itself. One exception is an Australian cancer care coordination model that was empirically derived from focus group and interview data with cancer patients (n = 20), their unpaid caregivers (n = 4) and clinicians (n = 29). Seven components were identified in the Australian coordination model: 1) care organization; 2) access to and navigation through the healthcare system; 3) key worker allocation; 4) effective communication and cooperation among multidisciplinary teams, and with other health service providers; 5) complementary and timely service delivery; 6) adequate and timely information for the patient; and 7) needs assessment [8].

There are clear similarities between the Australian model and our new CoG model. Similarities include an emphasis on system design and navigation, cooperation between individual staff and professional groups, and the timely delivery of services and information. Differences are also apparent. The emphasis on the keyworker role that was evident in the Australian model is not as prominent in our British model. In contrast, the quality and characteristics of staff, a need to know who was in charge and who to trust and rely upon, and who might act as an advocate featured more prominently in the British data. This means the relational qualities of the staff member were stressed more in the British findings. This relational aspect places a firm emphasis on the qualities of staff in Britain rather than only on their individual professional role. The need to acknowledge the voluntary contributions that patients and unpaid caregivers provide was also stressed in our findings, but not in the Australian model. Another difference was the emphasis on efficient, centralized and yet flexible systems in the British data, and the need for this to be operationalized across social and health care settings. The gap between the provision of social and health care in the UK has been identified previously [36], and this is reinforced by our results.

### Factors that influence coordination

The idea that coordination is influenced by factors has previously appeared in an adapted coordination version of Donabedian’s model [37], and our findings build upon this by revealing a classification of types of influencing factors. We provide a detailed account of five factors that should be considered in relation to cohesive and coordinated service planning, commissioning and configuration. Our data highlights that adequate time for clinical tasks is essential for the delivery of care coordination. It also reveals that there is a need to educate staff regarding whether or not coordination forms part of their role, and how staff can help coordinate care with others. The data also shows that care teams need to be adequately staffed and interoperable technologies need to be instituted for coordination to be delivered. Patients and their unpaid caregivers are helped by health and social care systems that communicate, that are easy to understand and that work together. An example of one complex coordination intervention that involves an interoperable IT platform to aid communication across many service providers is *Coordinate My Care. Coordinate My Care* is currently being implemented in London and it also allows for patients to have their own record of care [5]. This acknowledges patients’ and unpaid caregivers’ need for accurate and timely information based on their preferences, circumstances, needs and abilities, while also acknowledging their roles as partners in care provision and coordination. CMC also acknowledges the valuable financial contribution that patients and caregivers make. For patients with long-term neurological conditions in the UK, the financial contribution of families has been estimated to be four times the amount (£82,620) of formal care provided by staff (£18,117) [38].

### Future considerations and recommendations

Our pre-clinical, phase I findings highlight the importance of developing and testing complex coordination interventions through a programmatic approach to research, and it illustrates the complexity of taking into account context when evaluating complex interventions that operate on an individual- and system-level. Accordingly, with reference to the Medical Research Council (MRC) framework for the development and evaluation of complex interventions [39], [40], we propose that the relationships between the information, relational and structural core components of our model now be examined in future research. A discrete choice experiment (DCE), although relatively novel in healthcare, could allow for coordination costs and patient preferences to be better known. A DCE could also help distinguish the inter-relationship between the model’s elements and influencing factors. Also, a phase II feasibility study to investigate a coordination intervention, informed by the CoG model, may have the potential to advance the development and evaluation of coordination interventions. Plus, a natural experiment of new interventions that are currently being implemented in the National Health Service could be conducted using outcome measures that correspond with this model. Measures that correspond with the elements in the CoG model may offer an alternative to the current overreliance on care satisfaction outcomes and process measures [34] as proxy measures for coordination, and these should be explored in phase II studies. Furthermore, our research may prompt measurement considerations for the US national coordination measurement atlas [41]. Our conceptual fidelity may also help refine the metrics associated with the NHS Outcomes Framework, especially as the framework focuses on improving the quality of life for those with long-term conditions (dimension two), and improving patient’s experiences of care (dimension four) [42]. In the long-term, our results may even have implications for what type of coordination activity should be linked with fiscal incentives [34] and who gets paid for coordinating care, especially as unpaid caregivers currently seem to contribute a great deal to coordinating care. In essence, this study is therefore a novel and important contribution to the international literature regarding coordination and it prompts considerations related to coordination research, fiscal incentives and measurement.

### Strengths and limitations of the study

Our study was a multi-perspective, qualitative, longitudinal study involving a case-study approach. It resulted in an empirically derived model of care coordination. The strengths of the study include the quality indicators embedded within the design (such as core and regular research meetings to aid consistency, researcher triangulation, memos and visual mapping for analysis), the use of a sampling matrix informed by purposive sampling, the collection of data from contrasting British settings and serial interviews to collect data over time. The case-study approach also enabled a detailed investigation of contextual factors that influence coordination. This study fulfils all of the 32 consolidated criteria for reporting qualitative studies [43]. This is an additional strength of the research because these criteria aid reporting transparency and therefore critical appraisal of the study.

Study limitations include the lack of observational data to enhance the trustworthiness of the findings. Also, a loss of data specificity probably occurred through the ongoing inductive thematic analysis of the cases to produce one coherent model. However, this ongoing analysis resulted in one coherent model applicable across care providers and settings, and this could not have been achieved without some loss of specificity. The inclusion of the findings for each case in Tables S1–S3 helps ensure credibility and transparency, and allows readers to review the primary findings for each case. We are also aware that we did not use member checking to enhance or check our findings with the respondents. We acknowledge that this may have been possible with a few of the patients that remained alive at the end of the study. Alternatively, our findings could have been checked or enhanced with further input from their unpaid caregivers or a separate more generic service-user group.

## Conclusions

Within the context of serious illness, care coordination is a shared responsibility and it is a complex intervention facilitated, in part, through the deliberate efforts of patients, unpaid caregivers and staff that work in partnership across services and settings. Our model addresses the paucity of care coordination models, it provides conceptual coordination fidelity, it provides an alternative to the current overreliance on process and satisfaction measures as proxy measures for coordination, and helps address the challenge of not oversimplifying a real-world problem, such as care coordination. Avoiding oversimplifying the real-world problem of care coordination in a complex health and social care system that caters for patients with complex needs is important to advance the development, evaluation and implementation of coordination interventions for patients and their unpaid caregivers in the future.

## Supporting Information

Table S1 **Summary of the findings for the combined assessment unit (Edinburgh, Scotland).**
(DOCX)

Table S2 **Summary of the findings for the general practice working in partnership with care homes (English Midlands, England).**
(DOCX)

Tables S3 **Summary of the findings for the three outpatient clinics in the academic health science center (London, England).**
(DOCX)
