# Supplementary material for: The Real-World Problem of Care Coordination: A Longitudinal Qualitative Study with Patients Living with Advanced Progressive Illness and Their Unpaid Caregivers
Source: PLoS One. 2014 May 2;9(5):e95523. doi: 10.1371/journal.pone.0095523 (PMC4008426; doi:10.1371/journal.pone.0095523)
Supplement: Table S1 — Summary of the findings for the combined assessment unit (Edinburgh, Scotland). (DOCX) [file pone.0095523.s001.docx]

**Table S1: Summary of the findings for the combined assessment unit (Edinburgh, Scotland)**

| *Coordination and illustrative quotes* | **Influencing factors** |
| --- | --- |
| *Knowledge and engagement:* Coordination is aided by knowledge of systems and professionals. It is compromised when this is lacking. “*I come from a social work background … I see so many isolated elderly people, who trust the system too much. Who aren’t able or don’t think they are allowed to challenge or even to ask the question: Why is this happening?” [CarerCAU113].* Knowing professionals’ roles and who is *“in charge”* of care helps *“They’re all running out saying I’m this, I’m that and I’m the next thing and you’re completely lost.” [PatientCAU001].* Engagement and knowledge improves coordination with identification of anomalies being more likely, and this helps improve outcomes and quality of care. | Transitions: defined as time points where a change in care delivery occurred or a transition to a new treatment needed to take place. |
| *Staff trust and efficiency:* Trust is crucial to coordination. *“There is a certain trust that the medics know what they are doing, and then at some point, someone…will give the information that is needed.” [CarerCAU113].* In addition to the trust that is formed, enabled through the provision of standard care, trust in staff is gained through good communication, holistic care and the recognition of patient/unpaid caregiver vulnerability in relation to their illness experience. Trust in the system is eroded when “*waste”* is noticed. Patients and unpaid caregivers adapt by fighting their corner *“Years ago I wouldn’t have said boo to anybody … but I know now that you’ve got to, if you want something done, … stick up for yourself” [CarerCAU104]* and they learn to navigate the system and take control. *“It sometimes has to be challenged. You have to say to a doctor, did you not know?” [CarerCAU113]* |  |
| *Flexible and convenient care:* System flexibility is appreciated for appointments and emergency care options. Inflexible systems and inflexible care can fuel patients’ feelings of *“being a burden”* leading to disengagement from the system and questioning. “*[Staff sometimes say]...please don’t make an appointment unless it is absolutely necessary. Well, what do you know is absolutely necessary? That’s my argument. I never send for a doctor….I could be practically dying before I’ll send for a doctor.” [PatientCAU020]* |  |
| *Clinical environments:* Environments shape impressions of coordination. Negative environments are associated with poor care, lack of professionalism and coordination. Positive environments are associate with appreciation and value. *“Before when I was in hospital they never changed the beds every day.… these three times I’ve been in, everything’s been changed every day and I thought that’s amazing… that was for our comfort… very much appreciated…everything’s kept well in my ward.” [PatientCAU020]* |  |
