# Supplementary material for: The Real-World Problem of Care Coordination: A Longitudinal Qualitative Study with Patients Living with Advanced Progressive Illness and Their Unpaid Caregivers
Source: PLoS One. 2014 May 2;9(5):e95523. doi: 10.1371/journal.pone.0095523 (PMC4008426; doi:10.1371/journal.pone.0095523)
Supplement: Table S2 — Summary of the findings for the general practice working in partnership with care homes (English Midlands, England). (DOCX) [file pone.0095523.s002.docx]

**Table S2: Summary of the findings for the general practice working in partnership with care homes (English Midlands, England)**

| *Coordination and illustrative quotes* | **Influencing factors** |
| --- | --- |
| *High-quality care and staff with clear roles, relationships and adequate resources***:** High-quality care, and high-quality, well-informed, reliable and adequately resourced services and staff with clear roles and relationships that care, communicate and can work with and alongside other staff, patients and unpaid caregivers aid coordination. *“I believe that [X] may have spoken to [Y] but I’m not sure so I’m just wondering whether something has gone amiss, particularly since the person I spoke to had not heard of [Y] who is presumably an extremely prominent member of their staff.” [PatientGP012]* | Three influencing factors were identified: adequate resources, temporal constraints (e.g., not having enough time to do your job) and communication across settings. |
| *Responsive, logical and simple systems centered on patients and unpaid caregivers:* Responsive, non-mechanical, transparent, logical and simple systems based on patient and unpaid caregiver need, abilities and resources and systems that don’t lose people are essential to coordination. *“The point I’m making is that I went to see a doctor and they said I wasn’t their patient. I said ‘yes I am, I have been since I was that big’ and they said they didn’t have a record. Fortunately they had kept a record of [me in the archives].” [PatientGP011]* Systems that ensure continuity, good experiences and acknowledge patient and unpaid caregivers as partners in care are essential. |  |
| *Standardized, automated, reliable, cohesive systems and services with a personal touch:* Standardized, automated, joined-up systems with standardized delivery of care that involve contact with staff are essential. *“But every time I ring, when I eventually get through because apparently it goes through ambulance or fire places, I think it’s over in [city], and then it has to come back here [to my city], but the clinic is only down the road. If I could lift him and get him down to the nurse, and see here, he’s there, change it. But it doesn’t work like that you see. It’s all complicated now.” [CarerGP006]* The systems and services that are needed to treat people adequately must also involve a personal touch, double checks and transport to ensure appointments can be kept. *“People aren’t taking notice of what’s on the computer.” [PatientGP001]* Competent managers and leaders are vital to coordination. |  |
| *Timely, traceable, accurate and useful information with consideration of the implications of this information on unpaid caregivers:* Timely and tailored delivery of optimal amounts of accurate and useful information and paperwork that is traceable is required. An optimal numbers of services for different patient and caregiver groups can be useful for coordinating care. The implications for unpaid caregivers should be considered as this will aid care coordination. *“At [Z] hospital, when she had breast cancer and she had a kidney out of course, the right kidney out, they would ring you and keep you up to date.” [PatientGP009]* |  |
| *Unpaid caregivers and patients coordinate care, but at a cost:* Unpaid caregivers and patients are essential to coordination, but this is sometimes contributed at a personal cost. Unpaid caregivers and patients are the ones who often remember who needs to be where and why. *“I have to look on the calendar to see if we’re doing anything. I know Wednesday he has to have his injection and the following day he has to give blood and the day at the end of the month…” [CarerGP003].* |  |
