# Supplementary material for: The Real-World Problem of Care Coordination: A Longitudinal Qualitative Study with Patients Living with Advanced Progressive Illness and Their Unpaid Caregivers
Source: PLoS One. 2014 May 2;9(5):e95523. doi: 10.1371/journal.pone.0095523 (PMC4008426; doi:10.1371/journal.pone.0095523)
Supplement: Tables S3 — Summary of the findings for the three outpatient clinics in the academic health science center (London, England). (DOCX) [file pone.0095523.s003.docx]

**Table S3: Summary of the findings for the three outpatient clinics in the academic health science center (London, England)**

| **Categories with illustrative quotes** | **Influencing factors** |
| --- | --- |
| *Professional characteristics:* Experienced and committed professionals with excellent interpersonal skills aid care coordination. *“*S*he always asked me how I was .... Listened to my chest… kept a general eye on me … if I needed anything, she used to sort it out.” [PatientClinic008]*  When these qualities were lacking, it seemed as though staff didn’t care or that maybe they were incompetent. “*But when they don’t call…what do I make of that? Do I think she doesn’t care?” [CarerClinic001]* | Six influencing factors emerged: 1) management systems, efficiency and decisions; 2) personnel and temporal resources; 3) staff focus, experience and working relationships; 4) care based on need rather than diagnosis; 5) patients’ views and wishing to not burden the system or staff; 6) system clarity. |
| *Interconnected service structures and IT systems:* Multidisciplinary services that are simple with a central contact or advocate, regular appointments and emergency contacts aid coordination. *“I don’t think that the system is fool-proof enough….[it] is overly complicated and it’s so open to errors.” [CarerClinic001]* Services that are automated yet flexible and have integrated IT systems are integral to coordination. *“Within the hour…they phoned me “’Yeah, it’s ready you can come and collect it; do you want me to send it to the chemist...?’ Really good... it’s much smoother…” [CarerClinic005]* |  |
| *Patient empowerment, knowledge and experience, and ways to avoid waste:* Well-informed patients who are included in all communication significantly contribute to care coordination. *“The patient has got a right to know. One doctor, this one doctor to that doctor, that doctor to that doctor, that middle patient should be involved.” [PatientClinic016]* Care coordination is helped when patients feel listened to, respected and acknowledged as experts of their condition, and when their time, knowledge and resources are respected. This avoids waste. |  |
| *Recognition of carers as coordinators:* The formal integration of unpaid caregivers into care processes, knowledge exchange, and being respected and appreciated by professionals is essential to coordination. Relying on unpaid caregivers results in a temporal commitment, emotional resources and lost opportunity costs. *“There’s so much taken for granted that the family can do. It annoys me because if I wasn’t here they would have to do something....they don’t [offer help] unless you ask for it, and even then they don’t want to do it. [CarerClinic001]* |  |
